# Should Adrenal Venous Sampling Be Performed in PA Patients Without Apparent Adrenal Tumors?

**DOI:** 10.3389/fendo.2021.645395

**Published:** 2021-04-12

**Authors:** Kentaro Okamoto, Youichi Ohno, Masakatsu Sone, Nobuya Inagaki, Takamasa Ichijo, Takashi Yoneda, Mika Tsuiki, Norio Wada, Kenji Oki, Kouichi Tamura, Hiroki Kobayashi, Shoichiro Izawa, Akiyo Tanabe, Mitsuhide Naruse

**Affiliations:** ^1^ Department of Diabetes, Endocrinology and Nutrition, Kyoto University Graduate School of Medicine, Kyoto, Japan; ^2^ Division of Metabolism and Endocrinology, Department of Internal Medicine, St. Marianna University School of Medicine, Kawasaki, Japan; ^3^ Department of Diabetes and Endocrinology, Saiseikai Yokohamashi Tobu Hospital, Yokohama, Japan; ^4^ Department of Health Promotion and Medicine of the Future, Graduate School of Medical Sciences, Kanazawa University, Kanazawa, Japan; ^5^ Department of Endocrinology and Metabolism, National Hospital Organization Kyoto Medical Center, Kyoto, Japan; ^6^ Department of Diabetes and Endocrinology, Sapporo City General Hospital, Sapporo, Japan; ^7^ Department of Molecular and Internal Medicine, Graduate School of Biomedical and Health Sciences, Hiroshima University, Hiroshima, Japan; ^8^ Department of Medical Science and Cardiorenal Medicine, Yokohama City University Graduate School of Medicine, Yokohama, Japan; ^9^ Division of Nephrology, Hypertension and Endocrinology, Nihon University School of Medicine, Tokyo, Japan; ^10^ Division of Endocrinology and Metabolism, Tottori University Faculty of Medicine, Yonago, Japan; ^11^ Division of Endocrinology, National Center for Global Health and Medicine, Tokyo, Japan; ^12^ Endocrine Center, Ijinkai Takeda General Hospital, Kyoto, Japan

**Keywords:** adrenalectomy, adrenal venous sampling, cardiovascular disease, hyperaldosteronism, primary aldosteronism

## Abstract

**Introduction:**

Some aldosterone-producing micro-adenomas cannot be detected through image inspection. Therefore, adrenal venous sampling (AVS) is often performed, even in primary aldosteronism (PA) patients who have no apparent adrenal tumors (ATs) on imaging. In most of these cases, however, the PA is bilateral.

**Objective:**

To clarify the clinical need for AVS in PA patients without apparent ATs, taking into consideration the rates of adrenalectomy.

**Methods:**

This is a retrospective cross-sectional study assessing 1586 PA patients without apparent ATs in the multicenter Japan PA study (JPAS). We analyzed which parameters could be used to distinguish unilateral PA patients without apparent ATs from bilateral patients. We also analyzed the prevalences of adrenalectomy in unilateral PA patients.

**Results:**

The unilateral subtype without an apparent AT was diagnosed in 200 (12.6%) of 1586 PA patients. Being young and female with a short hypertension duration, normokalemia, low creatinine level, low plasma aldosterone concentration, and low aldosterone-to-renin ratio (ARR) was significantly more common in bilateral than unilateral PA patients. If PA patients without apparent ATs were female and normokalemic with a low ARR (<560 pg/ml per ng/ml/h), the rate of unilateral PA was only 5 (1.1%) out of 444. Moreover, 77 (38.5%) of the 200 did not receive adrenalectomy, despite being diagnosed with the unilateral subtype based on AVS.

**Conclusion:**

The low prevalence of the unilateral subtype in PA patients without apparent ATs suggests AVS is not indicated for all of these patients. AVS could be skipped in female normokalemic PA patients without apparent ATs if their ARRs are not high. However, AVS should be considered for male hypokalemic PA patients with high ARRs because the rates of the unilateral subtype are high in these patients.

## Introduction

Primary aldosteronism (PA) is characterized by inappropriate aldosterone production leading to renin suppression, which, if prolonged and severe, may in turn lead to hypertension and hypokalemia. The reported prevalence of PA varies, but recent studies suggest it accounts for about 6% of patients with hypertension (1). The etiologies of PA include two main subtypes diagnosed through adrenal venous sampling (AVS): unilateral, which is often treated surgically, and bilateral, which is most often treated medically (2). Aldosterone is reported to do substantial damage to cardiovascular organs under conditions of high salt intake (3), and previous clinical studies have reported that the prevalence of cardiovascular disease (CVD) was higher in PA patients than patients with essential hypertension (4). In particular, among patients with unilateral PA, which is often caused by an aldosterone producing adenoma, the prevalence of CVD is reportedly higher than among those with bilateral PA (4). And because unilateral PA patients can be treated with adrenalectomy, a diagnosis of PA subtype is relevant.

However, aldosterone-producing micro-adenomas are not always detected through image inspection, necessitating the use of AVS (5, 6). Although AVS is the most reliable method for accurate determination of PA subtype, it has several limitations, including its high cost, invasiveness, associated radiation exposure, and technical difficulty (7–10). On the other hand, probably because PA screening has become more popular, AVS is often performed proactively, even in PA patients without resistant hypertension, a high plasma aldosterone concentration (PAC) or hypokalemia. Consequently, the rate at which PA patients are being diagnosed with unilateral PA on AVS is on the decline and is only about 30% in the multicenter Japan PA study (JPAS) database (11). There is thus a need to be cautious about the indication for AVS in PA patients, especially those without ATs.

In the present study, we analyzed which parameters could be used to distinguish unilateral PA patients without ATs from bilateral patients. We also assessed the rate of adrenalectomy in unilateral PA patients.

## Materials and Methods

### Study Design and Patients

This study was conducted as a part of the JPAS and was a retrospective cross-sectional analysis. A nation-wide PA registry has been established at 40 centers in Japan, including 21 university hospitals and 19 city hospitals. We used a dataset valid as of March 2019. PA patients who were diagnosed and underwent AVS between January 2006 and January 2019 were enrolled. Patients eligible for enrollment in the JPAS were men and women aged 20-90 years. Patients whom the investigators deemed unsuitable were excluded. The clinical characteristics, biochemical findings, results of confirmatory testing, imaging findings, AVS results, treatments, surgical findings, and related follow-up data were electronically collected using the WEB registry system. System construction, data security, and maintenance of the registered data were outsourced to EPS Corporation (Tokyo, Japan). The data that support the findings of this study are available from the corresponding author upon reasonable request.

From among 4050 PA patients, 1586 were ultimately included in the present study. The reasons for exclusion of the other 2464 patients were as follows: the patient lacked imaging data or the imaging revealed an apparent AT (n=2099); AVS was without ACTH stimulation or was unsuccessful, which means there was a low selectivity index or no data on the selectivity index (n=285); or there was not enough data for analysis (n=80).

### Diagnosis of PA

The diagnosis of PA was made in accordance with guidelines from the Japan Endocrine Society and the Japan Society of Hypertension (12, 13). PA was diagnosed based on positive case detection when the ratio of the PAC (measured in pg/mL) to the plasma renin activity (PRA) (measured in ng/mL per h) was greater than 200, or when the ratio of the PAC to the active renin concentration (ARC) (measured in pg/mL) was greater than 40 and there was a positive result from at least one confirmatory test, such as the captopril-challenge test, saline-infusion test, furosemide-upright test, or the oral salt-loading test. Antihypertensive medications were usually changed to Ca^2+^ channel blockers and/or α−adrenergic blockers, as appropriate, until a final diagnosis was made.

### Subtype Diagnosis

A diagnosis of the PA subtype was made based on an AVS with ACTH (Cosyntropin) stimulation, the procedures for which have been described elsewhere (14). Adrenal vein cannulation was defined as successful if the selectivity index was greater than five (6). The selectivity index was defined as the ratio of the cortisol concentration in the adrenal vein to that in the inferior vena cava. The unilateral PA subtype was diagnosed when the lateralization index was greater than four. The bilateral PA subtype was diagnosed when the lateralization index was equal to or lower than four. The lateralization index was calculated by dividing the aldosterone-to-cortisol ratio on the dominant side by that on the non-dominant side.

### Adrenal Lesions on Image Inspection

The findings on CT scans were evaluated by radiologists at each institution. An apparent nodule on a CT scan was deemed to be a nodule if it was greater than or equal to 10 mm in diameter following a previous report (15). The appearance was determined to be bilateral normal if the size of the nodule or thickness of adrenal gland was less than 10 mm on both sides.

### Measurements

We collected data on age, sex, smoking habits, drinking habits, hypertension duration, body mass index (BMI), systolic blood pressure (SBP), diastolic blood pressure (DBP), proportion taking oral K^+^, and various blood tests (K^+^, creatinine, estimated glomerular filtration rate (eGFR), aldosterone-to-renin ratio (ARR), PAC, PRA, fasting blood sugar (FBS), HbA1c (NGSP), total cholesterol (TC), triglyceride (TG), high density lipoprotein (HDL) and low density lipoprotein (LDL)). eGFR was calculated using the following equation: eGFR (mL/min/1.73m^2^) = 194 x serum creatinine^-1.094^ x age^-0.287^ x 0.739 (if female) (16). Low eGFR was determined as eGFR <60 mL/min/1.73m^2^. Hypokalemia was considered to be present if serum K^+^ was ≤3.5 mEq/L or when the patient was taking a K^+^ supplement at the diagnosis of PA. Oral K^+^ was administered if hypokalemia was present.

We also investigated the prevalences of clinical complications such as diabetes mellitus (DM), dyslipidemia, proteinuria, and CVD. Diagnoses of DM and dyslipidemia were made at each institution according to those guidelines (17, 18). The prevalence of proteinuria was defined as +, 2+ or 3+ protein in urinalyses. CVD included stroke (cerebral infarction, cerebral hemorrhage and subarachnoid hemorrhage), ischemic heart disease (myocardial infarction and angina pectoris), and heart failure requiring hospitalization for treatment. Stroke was confirmed by neurologists and ischemic heart disease was confirmed by cardiologists.

### Assay Methods

PACs were measured using commercially available radioimmunoassays or chemiluminescent enzyme immunoassays. The reference range for PACs with patients in a supine position was 30-159 pg/mL (SPAC-S Aldosterone kits, Fuji Rebio, Co., Ltd, Tokyo, Japan) at 37 centers and 30-159 pg/mL (Accuraseed Aldosterone, FUJIFILM Wako Pure Chemical Corporation, Osaka, Japan) at 3 centers. PRA was measured using a radioimmunoassay or enzyme immunoassay. The reference range for PRA with patients in a supine position was 0.3-2.9 ng/mL/h (PRA-FR RIA kits, Fuji Rebio, Co., Ltd, Tokyo, Japan) at 23 centers, 0.2-2.3 ng/mL/h (PRA EIA kits, Yamasa, Co., Ltd, Choshi, Japan) at 13 centers, and 0.2-2.7 ng/mL/h (PRA RIA kits, Yamasa, Co., Ltd, Choshi, Japan) at 3 centers. Plasma ARCs were measured using a chemiluminescent enzyme immunoassay (Accuraseed Renin (ARC), FUJIFILM Wako Pure Chemical Corporation, Osaka, Japan) at 1 center. The reference range for ARCs with patients in a supine position was 2.5-21.4 pg/mL.

### Statistical Analysis

Stata/SE ver. 14 software developed by StataCorp^®^ was used for statistical analyses. All data are expressed as the mean ± SD for normally distributed variables and as the median (25th to 75th percentile) for variables not normally distributed. Sex, smoking habits, drinking habits, proportion taking oral K^+^, hypokalemia, proteinuria, and histories of DM, dyslipidemia, and CVD were considered to be binary variables. Student’s *t* test or the Mann-Whitney U test was used to compare quantitative variables. Pearson’s χ^2^ test was used for categorical parameters. Cuzick’s non-parametric test was used for trends across ordered groups. Values of P <0.05 were considered to indicate significant differences. Logistic regression analysis was performed to determine which parameters correlated with unilateral PA after adjusting for the patients’ backgrounds. Odds ratios (ORs) for unilateral PA were expressed as OR ± 95% confidence interval. In the logistic regression analysis, we included parameters that significantly differed between unilateral and bilateral PA patients in the univariate analyses and did not significantly correlate with each other. We also checked the correlations among these parameters using the Spearman rank correlation test to avoid multicollinearity. We considered that multicollinearity could occur when the Pearson’s or Spearman’s correlation coefficient *r >*0.4 and P <0.05. In the analysis of accuracy of unilateral PA diagnosis, the optimal receiver operating characteristic (ROC) curve cutoff point for each parameter was determined using the maximum Youden index. The Youden index was calculated by subtracting 1 from the sum of the sensitivity and specificity.

### Ethics

The study was conducted in accordance with Declaration of Helsinki Guidelines and the guidelines for clinical studies published by the Ministry of Health and Labor, Japan, and was approved by the ethics committee of the National Hospital Organization Kyoto Medical Center as the project-leading center and by the institutional ethics committees of the participating centers. This observational study was registered as UMIN ID 18756. This study was performed using an opt-out methodology. The opt out option was presented on the website and as a notice in a prominent place at each center.

## Results

### Comparison of Bilateral and Unilateral PA Cases

**Figure 1:**
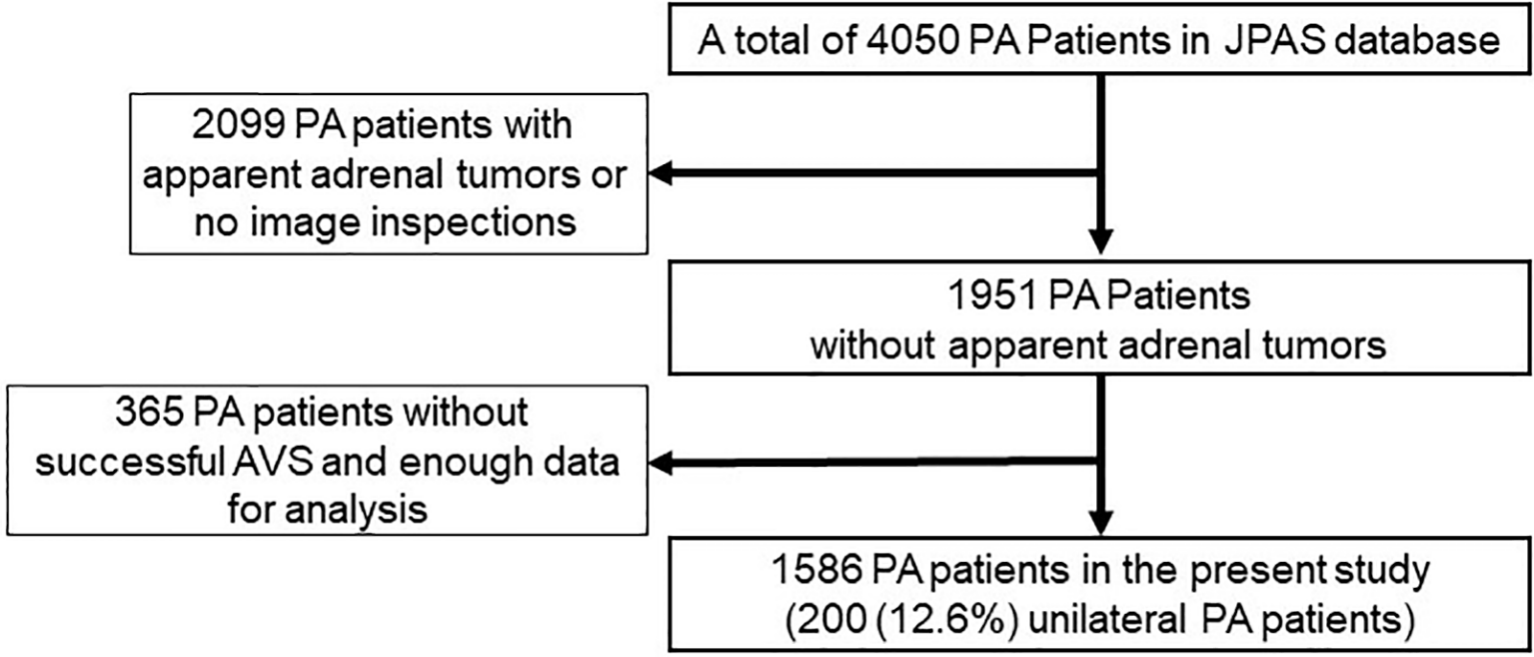
Patient selection criteria for inclusion in this study.

Among the 1,586 PA patients without apparent ATs enrolled in the JPAS study, 200 were diagnosed with unilateral PA and 1386 were diagnosed with bilateral PA (**Figure 1**; **Table 1**). There were more women in the bilateral PA group than in the unilateral PA group. In addition, significant differences were found between the two groups with respect to age, drinking habits, hypertension duration, serum creatinine level, TC, HDL and the rate of proteinuria. The prevalences of low eGFR and CVD were higher among unilateral than bilateral PA patients. SBP, DBP, history of smoking, TG, LDL, FBS, HbA1c, eGFR, and the rates of DM and dyslipidemia were similar between the two groups. Among the aldosterone-related parameters, PAC, ARR, and the rate of hypokalemia were significantly higher and the serum K^+^ level and PRA were significantly lower among unilateral than bilateral PA patients.

**Table 1:** Backgrounds and clinical complications of patients with bilateral and unilateral PA without apparent adrenal tumors.

Parameter	Bilateral PA patients n = 1386	Unilateral PA patients n = 200	P
Age, years	51.8 ± 10.9	53.7 ± 11.3	0.022*
Sex, male, %	43.4	71.5	<0.001*
Body mass index, kg/m^2^	25.2 ± 4.1	25.3 ± 4.1	0.784
History of Smoking, %	32.1	38.2	0.094
History of Drinking, %	52.0	61.3	0.017*
Systolic blood pressure, mmHg	142.1 ± 17.3	141.8 ± 18.3	0.792
Diastolic blood pressure, mmHg	87.9 ± 13.1	87.5 ± 13.0	0.676
Hypertension duration, years	3 (1-10)	10 (4-18)	<0.001*
Creatinine, mg/dl	0.73 ± 0.22	0.82 ± 0.23	<0.001*
EGFR, ml/min/1.73m^2^	79.4 ± 17.2	77.0 ± 21.1	0.087
Low eGFR, %	11.1	16.0	0.043*
Proteinuria, %	7.4	17.0	<0.001*
Total cholesterol, mg/dl	198 ± 33	188 ± 33	<0.001*
Triglyceride, mg/dl	132 ± 82	123 ± 66	0.184
LDL, mg/dl	116 ± 29	112 ± 29	0.112
HDL, mg/dl	57 ± 17	54 ± 15	0.024*
Dyslipidemia, %	27.9	34.1	0.089
Fasting blood sugar, mg/dl	106 ± 30	108 ± 29	0.446
HbA1c (NGSP), %	5.9 ± 1.0	5.8 ± 1.0	0.575
Diabetes mellitus, %	18.9	20.7	0.552
Serum K^+^, mEq/l	3.9 ± 0.4	3.5 ± 0.5	<0.001*
Hypokalemia, %	16.9	62.0	<0.001*
ARR, pg/ml per ng/ml/h	420 (290-660)	772 (427-1529)	<0.001*
PAC, pg/ml	156 (118-213)	228 (164-338)	<0.001*
PRA, ng/ml/h	0.4 (0.2-0.6)	0.3 (0.2-0.5)	<0.001*
Cardiovascular disease, %	6.2	10.0	0.045*

Student’s t test was used to compare parametric variables. The Mann-Whitney U test was used to compare nonparametric variables. Pearson’s χ^2^ test was used for categorical parameters. Asterisks (*) indicate significant differences (P <0.05). Hypokalemia was considered to be present if K^+^ was ≤3.5 mEq/L or when a patient was taking a potassium supplement. Low eGFR was defined as eGFR <60 ml/min/1.73m^2^. ARR, aldosterone-to-renin ratio; EGFR, estimated glomerular filtration rate; HDL, high density lipoprotein; LDL, low density lipoprotein; PA, primary aldosteronism; PAC, plasma aldosterone concentration; and PRA, plasma renin activity.

### Accuracy of Unilateral PA Diagnosis With Each Parameter

We next assessed the rates of unilateral PA, taking into consideration age, hypertension duration, serum creatinine level, serum K^+^ level, ARR, and PAC (**Table 2**). Cuzick’s non-parametric test for trend demonstrated that the prevalence of unilateral PA patients significantly increased as age, serum creatinine level, ARR or PAC increased or serum K^+^ decreased. We also used ROC curve analysis to calculate the optimal thresholds for these parameters. The optimal cutoff value in each parameter and its sensitivity, specificity, and likelihood ratio (LR) for distinguishing unilateral from bilateral PA are shown in **Table 3**. Serum K^+^, ARR and PAC had higher positive LRs than age, hypertension duration or serum creatinine level. Among categorical parameters, male and hypokalemia had low negative LRs, while hypokalemia, in particular, had the highest positive LR among these parameters.

**Table 2:** Rates of unilateral PA patients without apparent adrenal tumors in each parameter classified by quartile.

Parameter		Rates of Unilateral PA (%)	P
Age, years	<43	10.9	0.022*
	44-51	9.6	
	52-60	15.2	
	≥61	14.7	
HT duration, years	<1	5.5	<0.001*
	1-3	7.0	
	4-9	12.3	
	≥10	23.7	
Creatinine, mg/dL	<0.6	8.1	<0.001*
	0.6-0.70	8.2	
	0.71-0.83	12.3	
	≥0.84	21.6	
Serum K^+^, mEq/l	<3.6	34.2	<0.001*
	3.6-3.8	10.7	
	3.9-4.0	7.4	
	≥4.1	5.1	
ARR, pg/ml per ng/ml/h	<298	6.0	<0.001*
	298-442	7.1	
	443-742	11.2	
	≥743	26.2	
PAC, pg/ml	<123	2.6	<0.001*
	123-161	9.5	
	162-225	12.6	
	≥226	25.4	

Cuzick’s nonparametric test was performed to assess trends across ordered groups. Asterisks (*) indicate significant differences (P <0.05). ARR, aldosterone-to-renin ratio; HT, hypertension; PA, primary aldosteronism; and PAC, Plasma aldosterone concentration.

**Table 3:** Optimal cutoff values for each parameter and its sensitivity, specificity and likelihood ratio distinguishing unilateral PA patients without apparent adrenal tumors from bilateral PA patients.

Parameters	Optimal Cutoff Value	Sensitivity	Specificity	Positive Likelihood Ratio	Negative Likelihood Ratio
Age, years	55	51.5	60.1	1.29	0.81
HT duration, years	8	93.7	15.2	1.10	0.42
Creatinine, mg/dL	0.74	64.5	58.5	1.55	0.61
Serum K^+^, mEq/L	3.7	78.9	61.5	2.05	0.34
ARR, pg/ml per ng/ml/h	559	68.0	66.7	2.04	0.48
PAC, pg/ml	223	53.0	77.9	2.39	0.60
Sex, male	–	71.5	56.6	1.65	0.50
Low eGFR	–	16.0	88.9	1.44	0.94
Proteinuria	–	17.0	92.6	2.30	0.90
Hypokalemia	–	62.0	83.1	3.67	0.46
CVD	–	10.0	93.8	1.61	0.96

For continuous variables, the optimal cutoff value was determined using the maximum Youden index calculated from the ROC curve. Hypokalemia was considered to be present if K^+^ was ≤3.5 mEq/L or when a patient was taking a potassium supplement. Low eGFR was defined as eGFR <60 ml/min/1.73m^2^. ARR, aldosterone-to-renin ratio; CVD, cardiovascular disease; EGFR, estimated glomerular filtration rate; HT, hypertension; PA, primary aldosteronism; and PAC, plasma aldosterone concentration.

We also performed logistic regression analysis to determine which parameter could contribute to a diagnosis of unilateral PA after adjusting for variables showing significant differences in the univariate analyses (**Table 4**). All parameters were independent each other; however, because ARR potentially correlates with PAC and PRA, we selected age, sex, drinking habits, creatinine, hypokalemia, proteinuria, history of CVD, and ARR as independent variables. In this logistic regression analysis, male, long hypertension duration, hypokalemia, and high ARR significantly increased the odds of having unilateral PA; that means female, short hypertension duration, normokalemia, and low ARR were independently more common among bilateral PA patients than unilateral PA patients.

**Table 4:** Odds ratios and 95% confidence intervals for each parameter to distinguish unilateral PA patients without apparent adrenal tumors from bilateral PA patients.

Parameter	Odds ratio	Standard error	P	95% Coefficient Interval
Age, years	0.992	0.010	0.414	0.974-1.011
Sex, male	2.899	0.752	<0.001*	1.743-4.822
History of Drinking	1.147	0.237	0.506	0.765-1.720
Hypertension duration, years	1.045	0.012	<0.001*	1.022-1.069
Creatinine, mg/dL	1.092	0.648	0.882	0.342-3.491
Hypokalemia	5.028	0.994	<0.001*	3.413-7.407
ARR, pg/ml per ng/ml/h	1.001	0.0001	<0.001*	1.0005-1.001
Proteinuria	1.278	0.374	0.403	0.720-2.269
Cardiovascular disease	1.035	0.335	0.916	0.548-1.953

Asterisks (*) indicate significant differences (P <0.05). For the odds ratios, numerators are the odds in the unilateral PA group; denominators are the odds in the bilateral PA group. ARR, aldosterone-to-renin ratio and PA, primary aldosteronism.

### Efficiency of Diagnosing Unilateral PA Based on Patients’ Backgrounds

**Figure 2:**
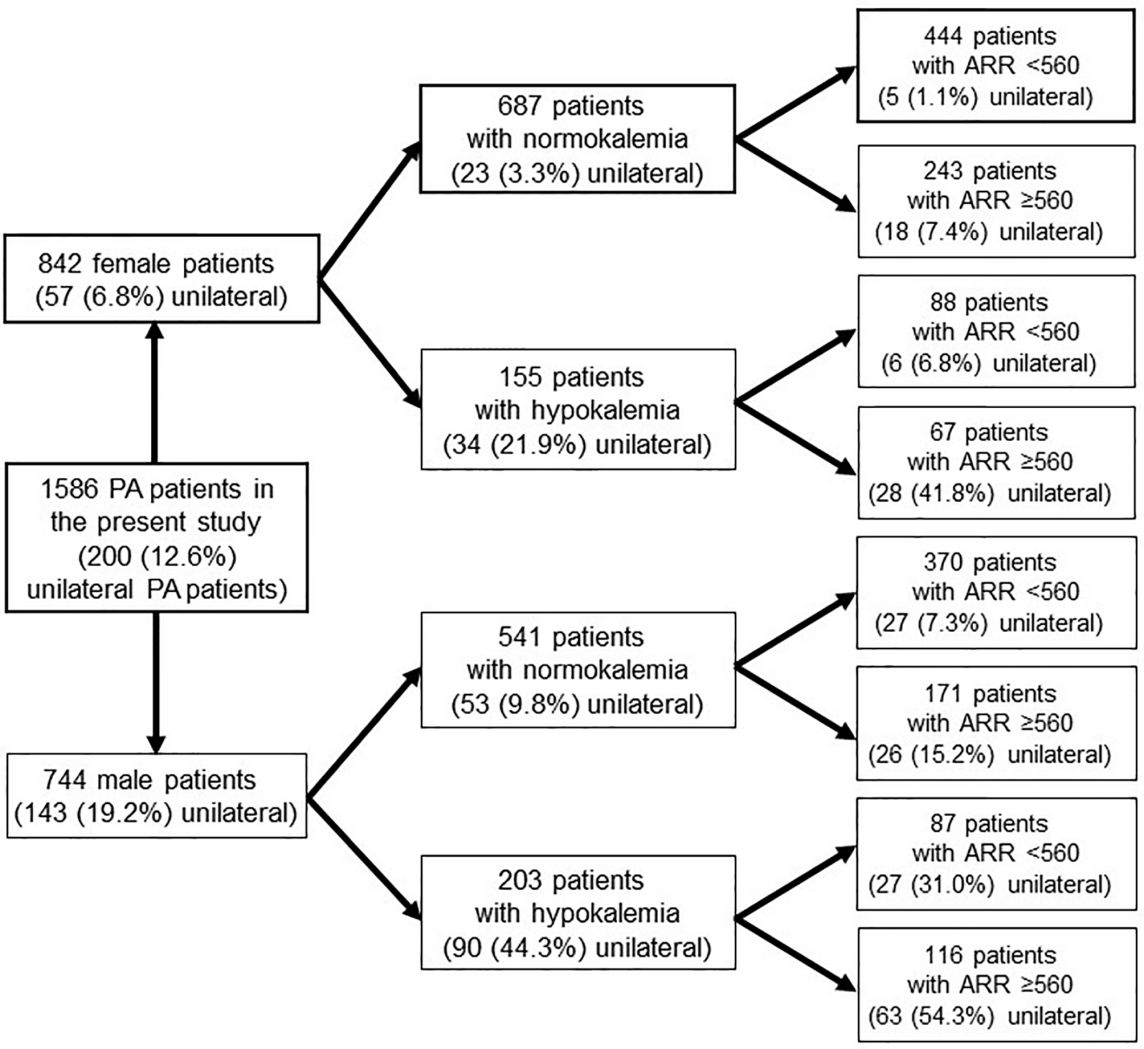
The prevalence of PA patients in each condition.

We considered whether we could efficiently distinguish unilateral PA patients without ATs from other patients based on the results of the logistic regression analysis (**Figure 2**). For this analysis, we did not select hypertension duration because it was unlikely that the reported duration was sufficiently accurate due to memory bias. When a PA patient without apparent ATs was female and did not exhibit hypokalemia, her rate of unilateral PA was only 3.3%. Moreover, the rate decreased to only 1.1% when her ARR was less than 560 pg/ml per ng/ml/h, which was the optimal cut-off with the maximum Youden index calculated from the ROC curve. In fact, only a single female unilateral PA patient presented with no apparent ATs and laboratory tests showing normokalemia and ARR less than 300 pg/ml per ng/ml/h. On the other, when a PA patient without apparent ATs was male and exhibited hypokalemia, his rate of unilateral PA was 44.3%, which increased 54.3% under the condition of ARR ≥560 pg/mL per ng/ml/h.

### Clinical Outcomes in Unilateral PA Patients Without Apparent ATs

Adrenalectomy was performed in 123 of the 200 PA patients without apparent ATs. After adrenalectomy, unilateral PA patients without apparent ATs who had postoperative data showed significant improvement in systolic BP (142.2 ± 17.5 to 130.2 ± 15.1, P<0.001), diastolic BP (87.7 ± 11.4 to 81.1 ± 10.7, P<0.001), serum K^+^ (3.4 ± 0.5 to 4.3 ± 0.3, P<0.001), and the number of antihypertensive drugs (1.35 ± 0.78 to 0.99 ± 0.80, P<0.001) after 6 to 12 months of follow-up. The pathological diagnoses in these 123 patients were as follows: 98 adenoma, 13 hyperplasia, 8 unable to determine whether adenoma or hyperplasia, 3 normal, and 1 unknown. On the other, unilateral PA patients treated with mineralocorticoid blockers also showed significant improvement in systolic BP (145.2 ± 19.3 to 136.4 ± 15.4, P=0.006) and serum K^+^ (3.6 ± 0.4 to 4.2 ± 0.5, P<0.001), but not in diastolic BP (87.2 ± 13.6 to 86.2 ± 11.5, P=0.590) after 6 to 12 months of follow-up.

## Discussion

In the present study, only 200 (12.6%) of the 1586 PA patients without apparent ATs were diagnosed with unilateral PA. Being young and female with short hypertension duration, normokalemia, a low creatinine level, low PAC, and low ARR was significantly more common in bilateral than unilateral PA patients. In particular, the rate of female normokalemic unilateral PA patients without apparent ATs and with ARRs less than 560 pg/ml per ng/ml/h was only 1.1%, and only a single patient (0.2%) had an ARR less than 300 pg/ml per ng/ml/h. Actually, hypokalemia, high PAC and high ARR were more common in unilateral than bilateral PA. This was especially true when PA patients without apparent ATs were in the highest quartile for PAC or ARR or in the lowest quartile for serum K^+^; for that group the rate of unilateral PA was close to 30%. In previous reports, sex, serum K^+^ level, eGFR, PAC, ARR and PAC after a captopril-challenge test or saline-infusion test, and CT findings for the adrenal glands were used to predict PA subtype (19–24). For example, studies by Umakoshi et al. and Kobayashi et al. emphasized normokalemic PA patients with bilateral normal results on CT were highly likely to be bilateral PA patients (23, 24). These findings suggest AVS may not be necessary to detect unilateral PA patients in all cases if we consider the patients’ backgrounds. In our study, the sensitivities and specificities of each parameter suggested sex, the serum K^+^ level, PAC and ARR could be helpful indicators when assessing the need for AVS in PA patients without apparent ATs. Therefore, among PA patients without apparent ATs, AVS is needed for male hypokalemic PA patients with a high ARR or PAC, given the high rates of the unilateral PA subtype. Conversely, AVS may be unnecessary for female normokalemic PA patients without high ARR due to the low rate of the unilateral subtype among that group.

A meta-analysis of PA patients has shown that, in Japan, men comprise a smaller proportion than do women. However, in other countries, men tend to comprise a higher proportion of PA patients than do women (25). Genetic mutations associated with development of aldosterone-producing adenomas have been reported (26). In particular, ATP1A1 and CACNA1D mutations have been found in men with small adenomas. In a recent study reported by Sam et al., about 90% of men with PA did not have apparent ATs (27). Therefore, in our study, the proportion of men could have appeared larger because we only investigated patients with unilateral PA patients without apparent ATs.

It has been reported that the unilateral PA subtype significantly increases the adjusted odds ratios for CVD, and the incidence of CVD among unilateral PA patients is significantly reduced by adrenalectomy as compared to medical treatment (4, 28). Furthermore, adrenalectomy was reported to lower blood pressure in PA patients, improve their quality of life, and reduce the incidences of DM and end stage renal disease as compared to medical treatment (29–32). It may therefore be desirable to treat unilateral PA patients with adrenalectomy, especially patients at high risk of CVD. In this study, we found that adrenalectomy was performed in 123 of the 200 PA patients without apparent ATs. In other words, 77 of the 200 unilateral PA patients without apparent ATs did not receive adrenalectomy, despite being diagnosed with the unilateral subtype. In Adrenal Venous Sampling Stats in Primary Aldosteronism (AVSTAT) study, about 70% of reasons for not undergoing adrenalectomy in unilateral PA patients depended on patient’s decision (33). In addition, some clinicians decided against adrenalectomy in patients with unilateral PA subtype because their blood pressure was well controlled, they were normokalemic, and they did not have definite adrenal tumors, most of these factors being known prior to AVS. In a recent study, 40 of 70 patients with AVS-confirmed unilateral PA and normal findings on adrenal imaging underwent adrenalectomy; this proportion is not high and similar to that found in our study (27). Even when their condition has been adequately explained, some patients choose not to undergo adrenalectomy after AVS. Another possible explanation for this low proportion is that improvements in systolic BP and hypokalemia were achieved by administering mineralocorticoid blockers to some unilateral PA patients without apparent ATs. However, the fact that adrenalectomy was not performed in about 40% of them suggests that for a substantial number of clinicians, there are barriers to surgically treating patients without apparent ATs unless patients have severe symptoms, like hypokalemia. In the present study, adrenalectomy resulted in significant improvement in hypertension and hypokalemia in unilateral PA patients, even those without apparent ATs. Sam et al. reported that 31 of 36 (86%) PA patients without apparent ATs had complete or partial responses (27). Therefore, adrenalectomy may be indicated in unilateral PA patients without apparent ATs.

Our study has several limitations. This is a retrospective cross-sectional study, which means the incidence of CVD among PA patients is unknown. We only evaluated the data from ACTH-stimulated AVS in this study, which means that bilateral or unilateral diagnostic results may differ in some cases when using the data from non-stimulated AVS. In this study, we defined adrenal lesions greater than or equal to 10mm as apparent nodules because it is difficult to detect lesions smaller than 10mm in some cases. Therefore, it cannot be ruled out that some patients in this study may have micro adenomas. In addition, imaging was not performed in all of our PA patients, which could introduce selection bias.

## Conclusion

The percentage of unilateral PA patients without apparent ATs was 12.6% and adrenalectomy was not performed in 38.5% of those patients, which suggests AVS is not necessary for all PA patients without apparent ATs. Especially in female PA patients without apparent ATs, AVS could be skipped if they do not have hypokalemia and high ARR. On the other hand, male PA patients with hypokalemia or a high PAC or ARR likely need AVS, given the high prevalence of the unilateral PA subtype among that group.

## Data Availability Statement

The datasets presented in this article are not readily available because the representative of this study is not the corresponding author. Requests to access the datasets should be directed to YO, bpm4567@kuhp.kyoto-u.ac.jp.

## Ethics Statement

The studies involving human participants were reviewed and approved by The ethics committee of the National Hospital Organization Kyoto Medical Center. Written informed consent for participation was not required for this study in accordance with the national legislation and the institutional requirements.

## Author Contributions

KOka, MN, MS, NI, and YO were involved in study design and data interpretation. KOka and YO were involved in the data analysis. AT contributed to create the calculable data. MT, TI, and TY especially collected a large amount of data and did data entry. KOki and KT checked the validity of statistical methods. HK, NW, and SI discussed the result and commented on the manuscript. MN, MS, and NI supervised the findings of this work. All authors contributed to the article and approved the submitted version.

## Funding

This study was conducted as a part of the JPAS (Japan PA Study) and JRAS (Japan Rare Adrenal Diseases Study) supported by research grants from the Japan Agency for Medical Research and Development (AMED) under grant numbers JP17ek0109122 (JPAS) and JP20ek0109352 (JRAS), and the National Center for Global Health and Medicine, Japan (27-1402, 30-1008). This study was also supported by grants from the Ministry of Health, Labor, and Welfare, Japan (Nanbyo-Ippan-046) and the Japan Smoking Foundation.

## Conflict of Interest

The authors declare that the research was conducted in the absence of any commercial or financial relationships that could be construed as a potential conflict of interest.

## References

[1] Käyser SC Dekkers T Groenewoud HJ van der Wilt GJ Carel Bakx J van der Wel MC. Study Heterogeneity and Estimation of Prevalence of Primary Aldosteronism: A Systematic Review and Meta-Regression Analysis. J Clin Endocrinol Metab (2016) 101(7):2826–35. 10.1210/jc.2016-1472 27172433

[2] Chao CT Wu VC Kuo CC Lin YH Chang CC Chueh SJ. Diagnosis and management of primary aldosteronism: an updated review. Ann Med (2013) 45(4):375–83. 10.3109/07853890.2013.785234 23701121

[3] Brown NJ. Contribution of aldosterone to cardiovascular and renal inflammation and fibrosis. Nat Rev Nephrol (2013) 9(8):459–69. 10.1038/nrneph.2013.110 PMC3922409 23774812

[4] Ohno Y Sone M Inagaki N Yamasaki T Ogawa O Takeda Y. Prevalence of Cardiovascular Disease and Its Risk Factors in Primary Aldosteronism: A Multicenter Study in Japan. Hypertension (2018) 71(3):530–7. 10.1161/HYPERTENSIONAHA.117.10263 29358460

[5] Kempers MJ Lenders JW van Outheusden L van der Wilt GJ Schultze Kool LJ Hermus AR. Systematic review: diagnostic procedures to differentiate unilateral from bilateral adrenal abnormality in primary aldosteronism. Ann Intern Med (2009) 151(5):329–37. 10.7326/0003-4819-151-5-200909010-00007 19721021

[6] Young WF Stanson AW Thompson GB Grant CS Farley DR van Heerden JA. Role for adrenal venous sampling in primary aldosteronism. Surgery (2004) 136(6):1227–35. 10.1016/j.surg.2004.06.051 15657580

[7] Rossi GP Barisa M Allolio B Auchus RJ Amar L Cohen D. The Adrenal Vein Sampling International Study (AVIS) for identifying the major subtypes of primary aldosteronism. J Clin Endocrinol Metab (2012) 97(5):1606–14. 10.1210/jc.2011-2830 22399502

[8] Monticone S Satoh F Dietz AS Goupil R Lang K Pizzolo F. Clinical Management and Outcomes of Adrenal Hemorrhage Following Adrenal Vein Sampling in Primary Aldosteronism. Hypertension (2016) 67(1):146–52. 10.1161/HYPERTENSIONAHA.115.06305 26573704

[9] Lubitz CC Economopoulos KP Sy S Johanson C Kunzel HE Reincke M. Cost-Effectiveness of Screening for Primary Aldosteronism and Subtype Diagnosis in the Resistant Hypertensive Patients. Circ Cardiovasc Qual Outcomes (2015) 8(6):621–30. 10.1161/CIRCOUTCOMES.115.002002 PMC4651757 26555126

[10] Fuss CT Treitl M Rayes N Podrabsky P Fenske WK Heinrich DA. Radiation exposure of adrenal vein sampling: a German Multicenter Study. Eur J Endocrinol (2018) 179(4):261–7. 10.1530/EJE-18-0328 PMC6182189 30299899

[11] Fujii Y Takeda Y Kurihara I Itoh H Katabami T Ichijo T. Historical changes and between-facility differences in adrenal venous sampling for primary aldosteronism in Japan. J Hum Hypertens (2020) 34(1):34–42. 10.1038/s41371-019-0229-4 31462725

[12] Nishikawa T Omura M Satoh F Shibata H Takahashi K Tamura N. Guidelines for the diagnosis and treatment of primary aldosteronism–the Japan Endocrine Society 2009. Endocr J (2011) 58(9):711–21. 10.1507/endocrj.EJ11-0133 21828936

[13] Shimamoto K Ando K Fujita T Hasebe N Higaki J Horiuchi M. The Japanese Society of Hypertension Guidelines for the Management of Hypertension (JSH 2014). Hypertens Res (2014) 37(4):253–390. 10.1038/hr.2014.20 24705419

[14] Umakoshi H Wada N Ichijo T Kamemura K Matsuda Y Fuji Y. Optimum position of left adrenal vein sampling for subtype diagnosis in primary aldosteronism. Clin Endocrinol (Oxf) (2015) 83(6):768–73. 10.1111/cen.12847 26123796

[15] Sherlock M Scarsbrook A Abbas A Fraser S Limumpornpetch P Dineen R. Adrenal Incidentaloma. Endocr Rev (2020) 41(6):775–820. 10.1210/endrev/bnaa008 PMC7431180 32266384

[16] Matsuo S Imai E Horio M Yasuda Y Tomita K Nitta K. Revised equations for estimated GFR from serum creatinine in Japan. Am J Kidney Dis (2009) 53(6):982–92. 10.1053/j.ajkd.2008.12.034 19339088

[17] Teramoto T Sasaki J Ishibashi S Birou S Daida H Dohi S. Diagnostic criteria for dyslipidemia. Diagn Criteria Dyslipidemia J Atheroscler Thromb (2013) 20(8):655–60. 10.5551/jat.17152 23892528

[18] Seino Y Nanjo K Tajima N Kadowaki T Kashiwagi A Araki E. Report of the committee on the classification and diagnostic criteria of diabetes mellitus. J Diabetes Investig (2010) 1(5):212–28. 10.1111/j.2040-1124.2010.00074.x PMC4020724 24843435

[19] Kupers EM Amar L Raynaud A Plouin PF Steichen O. A clinical prediction score to diagnose unilateral primary aldosteronism. J Clin Endocrinol Metab (2012) 97(10):3530–7. 10.1210/jc.2012-1917 22918872

[20] Nanba K Tsuiki M Nakao K Nanba A Usui T Tagami T. A subtype prediction score for primary aldosteronism. J Hum Hypertens (2014) 28(12):716–20. 10.1038/jhh.2014.20 24694802

[21] Kocjan T Janez A Stankovic M Vidmar G Jensterle M. A new clinical prediction criterion accurately determines a subset of patients with bilateral primary aldosteronism before adrenal venous sampling. Endocr Pract (2016) 22(5):587–94. 10.4158/EP15982.OR 26789347

[22] Kamemura K Wada N Ichijo T Matsuda Y Fujii Y Kai T. Significance of adrenal computed tomography in predicting laterality and indicating adrenal vein sampling in primary aldosteronism. J Hum Hypertens (2017) 31(3):195–9. 10.1038/jhh.2016.61 27582025

[23] Kobayashi H Abe M Soma M Takeda Y Kurihara I Itoh H. Development and validation of subtype prediction scores for the workup of primary aldosteronism. J Hypertens (2018) 36(11):2269–76. 10.1097/HJH.0000000000001855 30020243

[24] Umakoshi H Tsuiki M Takeda Y Kurihara I Itoh H Katabami T. Significance of Computed Tomography and Serum Potassium in Predicting Subtype Diagnosis of Primary Aldosteronism. J Clin Endocrinol Metab (2018) 103(3):900–8. 10.1210/jc.2017-01774 29092077

[25] Zhou Y Wang D Jiang L Ran F Chen S Zhou P. Diagnostic accuracy of adrenal imaging for subtype diagnosis in primary aldosteronism: systematic review and meta-analysis. BMJ Open (2020) 10(12):e038489. 10.1136/bmjopen-2020-038489 PMC7780716 33384386

[26] Dutta RK Söderkvist P Gimm O. Genetics of primary hyperaldosteronism. Endocr Relat Cancer (2016) 23(10):R437–54. 10.1530/ERC-16-0055 27485459

[27] Sam D Kline GA So B Pasieka JL Harvey A Chin A. Surgical Outcomes Among Primary Aldosteronism Patients Without Visible Adrenal Lesions. J Clin Endocrinol Metab (2021) 106(2):e824–35. 10.1210/clinem/dgaa821 PMC7823310 33180934

[28] Hundemer GL Curhan GC Yozamp N Wang M Vaidya A. Cardiometabolic outcomes and mortality in medically treated primary aldosteronism: a retrospective cohort study. Lancet Diabetes Endocrinol (2018) 6(1):51–9. 10.1016/S2213-8587(17)30367-4 PMC5953512 29129576

[29] Wu VC Chueh SJ Chen L Chang CH Hu YH Lin YH. Risk of new-onset diabetes mellitus in primary aldosteronism: a population study over 5 years. J Hypertens (2017) 35(8):1698–708. 10.1097/HJH.0000000000001361 28661412

[30] Velema M Dekkers T Hermus A Timmers H Lenders J Groenewoud H. Quality of Life in Primary Aldosteronism: A Comparative Effectiveness Study of Adrenalectomy and Medical Treatment. J Clin Endocrinol Metab (2018) 103(1):16–24. 10.1210/jc.2017-01442 29099925

[31] Katabami T Fukuda H Tsukiyama H Tanaka Y Takeda Y Kurihara I. Clinical and biochemical outcomes after adrenalectomy and medical treatment in patients with unilateral primary aldosteronism. J Hypertens (2019) 37(7):1513–20. 10.1097/HJH.0000000000002070 31145370

[32] Chen YY Lin YH Huang WC Chueh E Chen L Yang SY. Adrenalectomy Improves the Long-Term Risk of End-Stage Renal Disease and Mortality of Primary Aldosteronism. J Endocr Soc (2019) 3(6):1110–26. 10.1210/js.2019-00019 PMC6507624 31086833

[33] Ohno Y Naruse M Beuschlein F Schreiner F Parasiliti-Caprino M Deinum J. Adrenal venous sampling guided adrenalectomy rates in primary aldosteronism: results of an international cohort (AVSTAT). J Clin Endocrinol Metab (2020) 106(3):e1400-7. 10.1210/clinem/dgaa706 33031550

